# Data-Driven Decisions for Reducing Readmissions for Heart Failure: General Methodology and Case Study

**DOI:** 10.1371/journal.pone.0109264

**Published:** 2014-10-08

**Authors:** Mohsen Bayati, Mark Braverman, Michael Gillam, Karen M. Mack, George Ruiz, Mark S. Smith, Eric Horvitz

**Affiliations:** 1 Stanford University, Stanford, California, United States of America; 2 Princeton University, Princeton, New Jersey, United States of America; 3 MedStar Health and Washington Hospital Center, Washington, D. C., United States of America; 4 Microsoft Research and University of Washington School of Medicine, Redmond, Washington, United States of America; Old Dominion University, United States of America

## Abstract

**Background:**

Several studies have focused on stratifying patients according to their level of readmission risk, fueled in part by incentive programs in the U.S. that link readmission rates to the annual payment update by Medicare. Patient-specific predictions about readmission have not seen widespread use because of their limited accuracy and questions about the efficacy of using measures of risk to guide clinical decisions. We construct a predictive model for readmissions for congestive heart failure (CHF) and study how its predictions can be used to perform patient-specific interventions. We assess the cost-effectiveness of a methodology that combines prediction and decision making to allocate interventions. The results highlight the importance of combining predictions with decision analysis.

**Methods:**

We construct a statistical classifier from a retrospective database of 793 hospital visits for heart failure that predicts the likelihood that patients will be rehospitalized within 30 days of discharge. We introduce a decision analysis that uses the predictions to guide decisions about post-discharge interventions. We perform a cost-effectiveness analysis of 379 additional hospital visits that were not included in either the formulation of the classifiers or the decision analysis. We report the performance of the methodology and show the overall expected value of employing a real-time decision system.

**Findings:**

For the cohort studied, readmissions are associated with a mean cost of $13,679 with a standard error of $1,214. Given a post-discharge plan that costs $1,300 and that reduces 30-day rehospitalizations by 35%, use of the proposed methods would provide an 18.2% reduction in rehospitalizations and save 3.8% of costs.

**Conclusions:**

Classifiers learned automatically from patient data can be joined with decision analysis to guide the allocation of post-discharge support to CHF patients. Such analyses are especially valuable in the common situation where it is not economically feasible to provide programs to all patients.

## Introduction

The increasing availability of large quantities of clinical data being captured by electronic health record systems frames opportunities for generating predictive models from that data to enhance healthcare. We first describe the construction of a predictive model that forecasts the likelihood that heart failure patients will be rehospitalized within 30 days of their discharge. Then, we show how the predictive model can be coupled with an automated decision analysis, which uses patient-specific predictions about readmissions within a cost-benefit model, to provide guidance on the patient-specific allocation of readmission reduction programs. We discuss how such a coupling creates a valuable *data to prediction to action* pipeline that can be implemented within a decision-support system to guide interventions. We describe methods for employing a cohort of test patients, held out from the development of the predictive models, to characterize the overall value of using a real-time decision system given uncertainty in costs and efficacies. This approach provides a foundation for understanding the relationship between interventions that promise to reduce readmission rates at some cost and predictive models that provide forecasts about individual patients' readmission risk.

High rates of rehospitalization within 30 days of discharge reported at multiple health centers have motivated studies of the use of predictive models to identify patients at the highest risk for readmission and the feasibility of using these inferred likelihoods to guide clinical decision making. In a 2007 report to the U.S. Congress, the Medicare Payment Advisory Commission (MedPAC) indicated that as much as $12B is spent annually by Medicare on potentially preventable readmissions within 30 days of patient discharge [1]. Beyond monetary costs, readmission within a short time horizon may be an indicator of poor quality of care with implications for the quality and length of patient lives. Incentives that would encourage hospitals to reduce the rates of unnecessary readmissions have been proposed and are being implemented [2].

Numerous programs for reducing readmissions have been implemented and tested over the past twenty years [3]–[11]. These programs have sought reductions in readmissions via investments in post-discharge care coordination, patient education and self-management support, scheduled outpatient visits, and telemedicine. A recent survey of readmission reduction programs can be found in [12].

Post-discharge intervention studies described in the literature often report some reduction in readmission rates associated with the intervention being studied. Reported reductions in rates of readmission vary from a few percentage points to 50% or more. Reported per-patient intervention costs vary from a low of $180 [13]–[14] to $1,200 [15]. Results span savings of thousands of dollars per patient to reported net losses. [16].

Even when an intensive post-discharge program is found effective in preventing readmissions, it may be prohibitively expensive to provide such an intervention to an entire patient cohort. However, net savings may be achieved when the same interventions are applied in a selective manner to patients identified as being at high risk for readmission.

Some readmission reduction programs have been implemented as patient-specific, with enrollment of patients into the prevention program determined by risk scores [17]–[20]. Detailed surveys of such scores are provided in [21]–[22]. A notable example is LACE [17]. LACE and related scores are designed to be simple enough to be calculated manually at time of discharge.

We give an end-to-end demonstration of the methodology of per-patient cost-benefit analysis based on a risk score that is automatically inferred from EHR data. We perform a sensitivity analysis of the overall effectiveness of readmission reduction programs over reported ranges of efficacies and costs of interventions and probe the expected value of embedding a real-time system providing these automated inferences in the clinical workflow. Finally, we compare the utility of our score for the end-to-end process to that of LACE and show that even relatively modest improvements in prediction quality can make a substantial difference in the utility of an intervention.

The advances introduced include: (1) a means for harnessing data available in the local EHR to build the best possible predictive model based on the locally available data; (2) the ability to embed computational guidance on risk rather than relying on heuristic policies or manual calculations of risk scores; (3) the ability to use local financial data to perform a calibrated cost-benefit analysis that recommends an intervention based on the optimal expected payoff; and (4) the ability to perform offline analyses to probe the value of embedding real-time guidance on interventions in a clinical setting via exploring implications of different combinations of costs and benefits of the interventions. The results highlight the importance of combining predictions with decision analysis.

## Materials and Methods

We performed the studies on de-identified patient data drawn from the EHR system for a large tertiary urban hospital serving the Metropolitan Washington DC area. Approval for data access and analysis was granted by hospital's institutional review board.

The patient cohort consists of all Medicare patients who were admitted as inpatients to the hospital during July 1, 2007–June 30, 2010 and were discharged alive with heart failure as their primary diagnosis (ICD9 codes 402.01, 402.11, 402.91, 404.01, 404.03, 404.11, 404.13, 404.91, 404.93, and 428.x).

For each visit we defined a target binary variable to describe the patient's 30-day bounce-back status (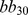). We defined all-cause rehospitalizations occurring within 30 days as 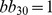 and no rehospitalization as 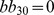. The correct value of 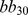 was obtained using data files provided by the U.S. Centers for Medicare & Medicaid Services (CMS) to the hospital in April 2011. Our goal was to estimate the probability of 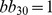 for patients where the value of 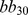 is unknown.

We divided the cohort into a derivation cohort, consisting of all visits that started between July 1, 2007–June 30, 2009, and a validation set of all visits that started between August 1, 2009–June 30, 2010. In order to build a predictive model, the following information was extracted from the EHR database for each patient:

- *Patient information*: age, gender, marital status, family support.
- *Visit information*: date, time, duration, type (inpatient, emergency, or outpatient), source (emergency, transfer, etc.).
- *Medical information*: history of diagnoses, lab results, medications, medical history, chief complaint, attending and admitting doctors.

For the analysis of costs, we gathered cost information for these visits from the hospital's administrative database. To be consistent with CMS definitions, we excluded the following visits that did not meet criteria for calculation of hospital readmission rates according to CMS 30-day mortality and readmission patient-level data files sent to the hospital.

- Patient was not enrolled in fee for service (FFS) Parts A and B (the standard U.S. Federal-funded health coverage for those over 65) during the 12 consecutive months prior to the index admission or in the 30 days after discharge.
- Patient died during the index hospitalizations.
- Patent left against medical advice (AMA).
- Patient was transferred to another acute care facility.
- Additional admission within 30 days of discharge from an index admission (an admission cannot be considered both an index admission and a readmission).

Our eligible cohort was reduced to 1,172 hospital visits including 793 visits in the derivation cohort and 379 visits in the validation group. Further refinement of the cohort to account for readmissions of patients to other medical facilities is presented in accompanied Text S1.

### Model-building and variable selection

We encoded the information about each visit as a vector of binary variables of size 3,388. The variables were extracted automatically from the patient visit data. We used logistic regression to compute probabilities of rehospitalization for cases in the validation set. Since our model contains a large set of variables relative to the cohort size, we use the LASSO technique to select the most predictive variables and avoid overfitting [23]–[24]. Overfitting is a challenge in statistics and machine learning that can specialize the predictive model to the training cases and provide predictors that do not ideally generalize to future, previously unseen cases. The optimal parameters of LASSO were selected based on a cross-validation analysis. The details of this analysis are described in the accompanied Text S1.

We also implemented LACE [17] in our cohort and compared its predictive accuracy with the statistical classifier using area under receiver-operator characteristic (ROC) curve and reclassification analysis. Details of this implementation and calibration of both LACE and the classifier is provided in the accompanied Text S1.

### From Predictions to Decisions

We coupled the predictive classifier for readmission with a decision model to perform patient-specific decision analyses for guiding post-discharge interventions. The decision model takes into account the predicted probability of readmission, cost and efficacy of applying the intervention, and average cost of readmission and allocates the interventions. We made the following simplifying assumptions:

- The cost of applying an intervention is on a per-patient basis and is the same for all patients. Most prior studies make this assumption. Interventions include assuring an outpatient visit within a specified time frame, rendering additional patient education, performing follow-up phone calls, and providing a fixed number of home visits.
- The expected cost 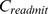 of readmission is *a priori* the same for all patients. 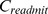 may include not just the monetary cost of a readmission, but also a penalty for the reduced quality of care associated with presentations warranting avoidable hospital readmissions. 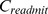 may be set by the hospital using a variety of considerations or just be calculated from past financial data. We obtained 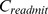 using the latter option by calculating the empirical average readmission costs for patients in the derivation cohort.
- The efficacy of the intervention is *a priori* the same 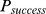 for the entire cohort of patients. For example, an intervention that reduces the readmission rate by 25% reduces the readmission risk of each patient to whom it is applied by 25%.

Without the intervention, the expected cost of readmission for a patient whose probability of being readmitted within 30 days is *p* is 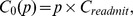
which is the full cost of a readmission, weighted by the likelihood of the readmission. The intervention adds 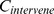 to the cost of treatment but promises to reduce the readmission probability to 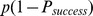, thus bringing the total expected cost to
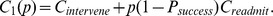



**Figure 1:**
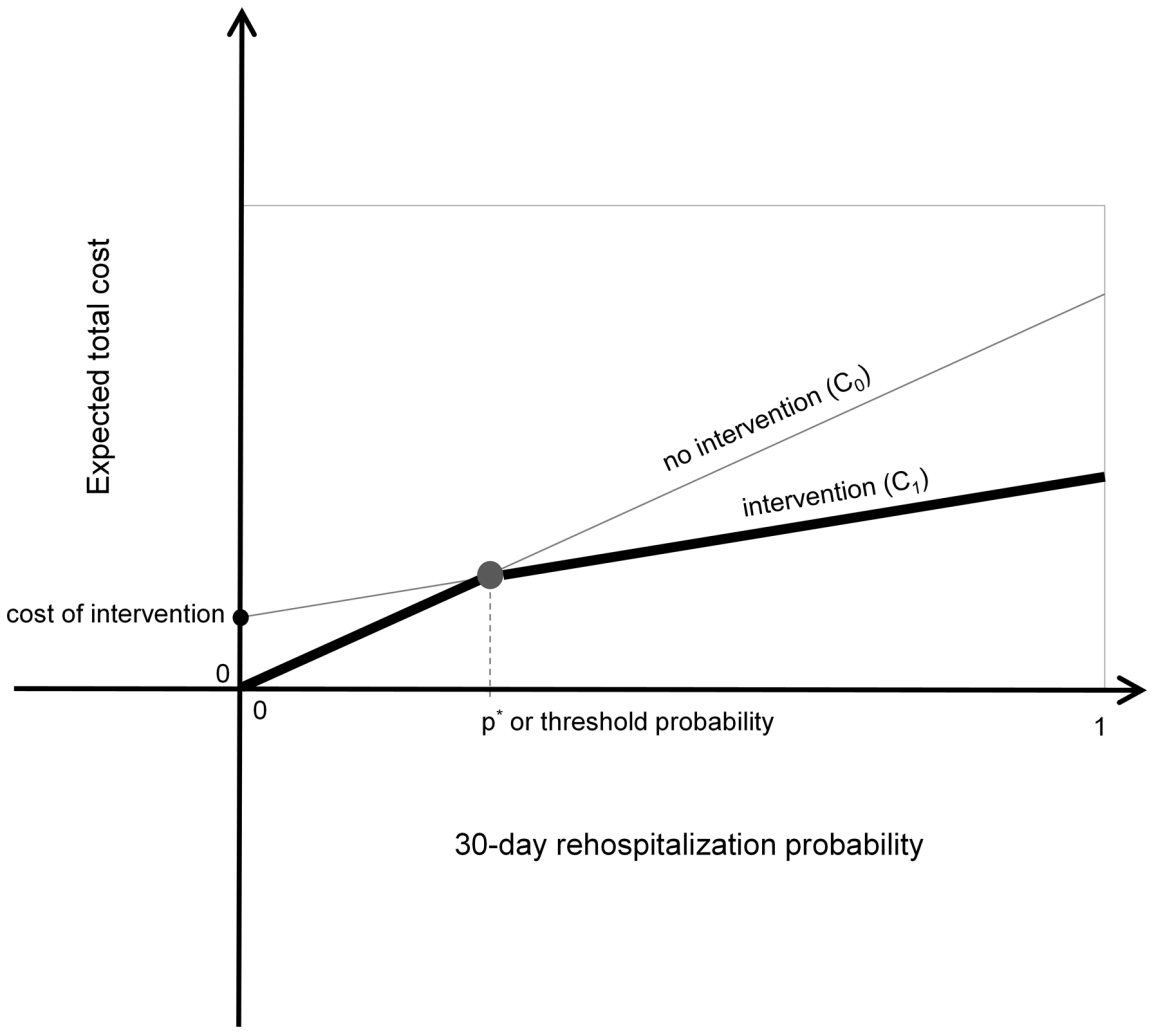
Plots of utility of outcomes for the intervention and no-intervention cases, showing relationship of potential expected utilities of outcomes achieved with a postdischarge program that reduces the rate of readmission versus the default of making no special intervention. Darker lines highlight ideal policy for any predicted likelihood of rehospitalization.



The expected cost of the outcomes associated with the cases of intervention and nonintervention as functions of *p* are plotted in **Figure 1**. The expected utility of the intervention is the difference of these expected values, 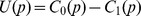. For low values of 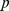, the intervention is not warranted, as 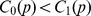 and 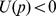. For high values of 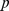, 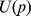 becomes positive and the intervention becomes beneficial. Note that the expected utilities associated with the intervention and default post-discharge programs cross at a threshold probability, which we denote by 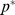. At this probability of readmission, the expected utility for the intervention and default programs are equal. We seek to maximize the overall benefit of the program for all patients by taking patient-specific actions that maximize the net expected benefit for each patient. The ideal policy for maximizing utility is to apply the intervention to patients for whom 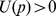, or equivalently

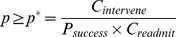



The predictive model provides patient-specific estimates of the readmission probability 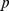 based on the available data, and we use the model to predict for each patient whether 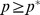.

Finally, to explore the importance of the accuracy of the classifier in this analysis, we repeated the study using the LACE score instead of the output of the learned classifier and compared the results of the two analyses.

### Software packages

All statistical analysis including construction of the classifier, cross-validation analysis, model calibration, and decision optimization was performed using R and Matlab.

## Results

### Model validation

The optimal classifier achieved an overall area under the curve (AUC) of 0.66 on the validation cohort. This is 10% improvement over the predictive model presented in [20], which obtained an AUC equal to 0.60, and an 11% improvement over LACE [17], which had an AUC of 0.59 in the validation cohort. The cross-validation AUC for the classifier and LACE for the derivation cohort is 0.69±0.0198 (p_value_ <0.05) and 0.59±0.0093 (p_value_ <0.05), respectively. The difference is statistically significant (p_value _<0.001). The fractions of readmissions to outside hospitals in the derivation and validation cohorts are 35.2% and 47.9%, respectively. This significant increase in outside readmissions highlights a major challenge of predicting readmissions in our data as the patient population and patterns of engagements with the hospital has significantly changed. If we remove from the validation cohort all patients who were readmitted to outside hospitals, the AUC of the classifier on the validation cohort is 0.71.

**Figure 2:**
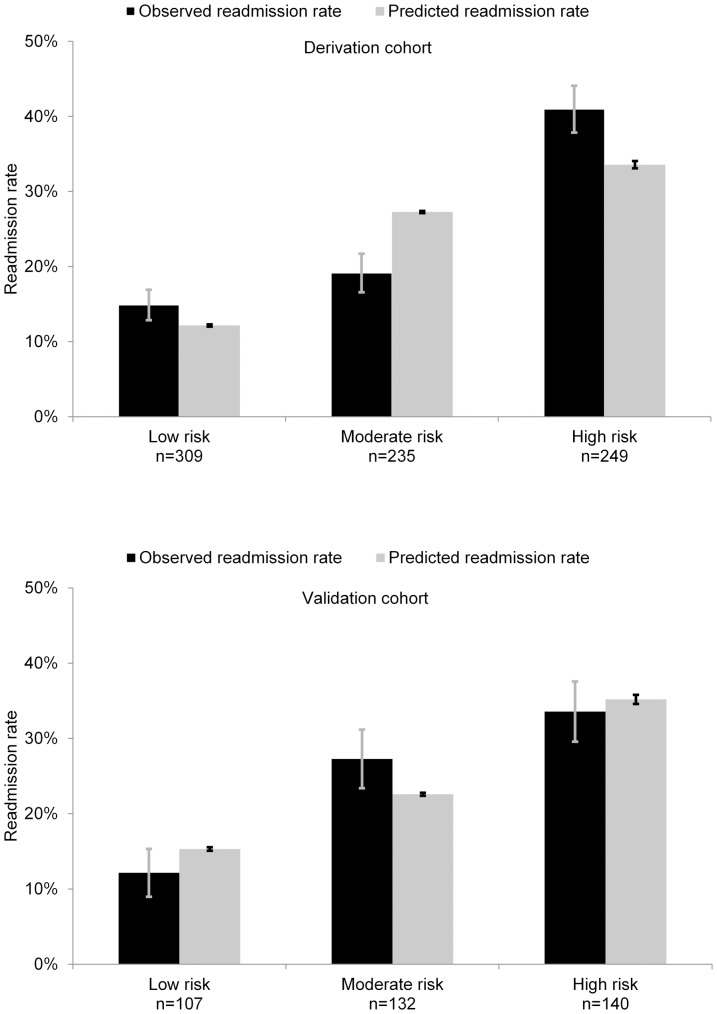
Study of calibration of likelihoods generated by predictive model. Figures show number of patients in each of three risk groups of derivation cohort (top) and validation cohort (bottom) with observed and predicted readmission rates within a margin of error with *p*_value_<0.05.

The observed and predicted probabilities of rehospitalization provided by the predictive model for both derivation and validation cohorts are shown in **Figure 2**. The reclassification of patients from the three risk groups obtained by LACE to the three risk groups obtained by the learned classifier is shown in **Table 1**.

**Table 1:** Reclassification of patients from the three risk groups obtained by LACE to revised risk groups obtained by the classifier.

Reclassification matrix					
		Classifier			
		Low risk	Moderate risk	High risk	Total reclassified (%)
**LACE**	**Low risk (%)**	35.8	36.2	28.0	**64.2**
	**Moderate risk (%)**	16.8	33.6	49.6	**66.4**
	**High risk (%)**	0.0	23.5	76.5	**23.5**

### Predictive variables

The optimal model automatically selects a subset of 253 variables from 3,388 available variables to avoid overfitting to the derivation data. **Tables 2** and **3** show lists of the variables identified as most influential in predicting rehospitalization within 30 days. The variables are separated into lists of findings that provide the most evidential support for (**Table 2**) and against (**Table 3**) a patient being rehospitalized and are clustered within each list into diagnosed diseases, medications, lab results, and patterns of engagement with the healthcare system. Observations on engagement include such statistics as time since the last hospitalization and the number of hospitalizations in the last year.

**Table 2:** Top variables selected by machine learning procedure that increase risk of readmission within 30 days.

Top supportive evidence			
Variable class	Variable description	Log Odds Ratio	Log Odds Ratio Standard Error^1^
Lab Results	Lymphocyte % is low	0.0128	0.0027
Patterns of Engagement	Patient was admitted in past 6 months	0.0112	0.0031
Lab Results	BUN is high	0.0038	0.0012
Lab Results	Glucose level random is elevated	0.003	0.0012
Lab Results	Monocyte absolute is low	0.0028	0.0012
Other Diagnoses	History of nondependent abuse of drugs (ICD9 305.x)	0.0018	0.001
Other Diagnoses	History of chronic airway obstruction, not elsewhere classified (ICD9 496.x)	0.0017	0.0008
Other Diagnoses	History of gastrointestinal hemorrhage (ICD9 578.x)	0.0014	0.0007
Lab Results	AST is elevated	0.0013	0.0006
Other Diagnoses	History of cardiomyopathy (ICD9 425.x)	0.001	0.0006
Lab Results	Magnesium is low	0.001	0.0006
Lab Results	INR is elevated	0.0009	0.0004
Patterns of Engagement	Patient has been in isolated room in hospital	0.0009	0.0006
Lab Results	BNP is high	0.0007	0.0005

These are variables that receive positive log-odds ratio with the largest magnitude.

1. Obtained from sample standard error for cross-validation odds ratios

**Table 3:** Top variables selected by machine learning procedure that decrease risk of readmission within 30 days.

Top disconfirming evidence			
Variable class	Variable description	Log Odds Ratio	Log Odds Ratio Standard Error^1^
Patterns of Engagement	Number of emergency room visits during past 6 months <2	−0.0607	0.0035
Lab results	Hematocrit % is normal	−0.0442	0.0043
Lab results	BNP is normal	−0.044	0.0049
Lab results	Alkaline phosphatase is normal	−0.0428	0.0033
Lab results	Chloride is normal	−0.0428	0.0042
Cardiac medications	Patient is not on digoxin therapy	−0.0396	0.0039
Lab results	MCHC % is low	−0.0387	0.0039
Changes in lab results	TSH variation during current visit is low	−0.0343	0.003
Changes in lab results	CO2 variation during current visit is low	−0.0318	0.0039
Changes in lab results	RDW variation during current visit is low	−0.0308	0.0038
Changes in lab results	MCV variation during current visit is low	−0.0306	0.0036

These are variables that receive negative log-odds ratio with largest magnitude.

1. Obtained from sample standard error for cross-validation odds ratios

### Exploring the value of a real-time decision system

The patient-specific decision analyses applied to each of the CHF patients in the validation cohort using the utility model described in the Materials and Methods section identifies patients in the validation cohort who would have received the post-discharge intervention according to a policy of minimizing expected care costs. We compared the efficacy of using such patient-specific decision analyses to the use of a simpler *uniform policy*, where interventions are allocated homogeneously to either all or none of the patients, based on an analysis of which of the two possible uniform policies (*apply to all* versus *apply to none*) has the highest net expected value.

**Table 4** compares the percentage of total readmission costs ($1,291,300) that would be saved, as predicted by the model, through using a patient-specific decision analysis as opposed to offering interventions to all patients in a uniform manner for efficacies (reduction in readmission rate) assumed to be 25%, 35% and for costs of intervention set to $800, $1,300, and $1,800. **Table 4** also shows the percentage of total expected readmissions prevented with the patient-specific analysis versus the best uniform policy. **Table 4** also displays the cost savings achieved by the patient-specific decision analysis when LACE is used in place of the statistical classifier. Using the poorer-performing LACE score for identifying patients at risk for readmissions translates into lower savings and narrower bands of usefulness across regimes defined by the costs and efficacies of programs.

**Table 4:** Comparison of savings achieved and readmissions prevented for post-discharge programs with different costs and efficacies.

Comparison of savings								
		Savings or losses				Readmissions prevented		
Cost of intervention	Efficacy	Patient-specific analysis and classifier	Patient-specific analysis and LACE	Intervention applied to all patients	Best uniform policy	Patient-specific analysis and classifier	Patient-specific analysis and LACE	Best uniform policy
$300	25%	16.2%	15.9%	16.2%	16.2%	25.0%	24.5%	25.0%
	35%	26.2%	26.2%	26.2%	26.2%	35.0%	35.0%	35.0%
$800	25%	5.4%	1.3%	1.5%	1.5%	17.4%	2.9%	25.0%
	35%	13.2%	9.1%	11.5%	11.5%	31.4%	22.2%	35.0%
$1,300	25%	0.7%	0.0%	−13.2%	0.0%	5.2%	0.0%	0.0%
	35%	3.8%	0.5%	−3.2%	0.0%	18.2%	2.6%	0.0%
$1,800	25%	0.3%	0.0%	−27.8%	0.0%	0.8%	0.0%	0.0%
	35%	0.8%	0.0%	−17.8%	0.0%	7.3%	0.0%	0.0%

Three policies are compared: patient specific analysis using the classifier, patient-specific analysis using LACE, best uniform policy. For comparison of savings an additional column demonstrates the policy that applies intervention to every patient may lead to loss (or savings).

We note that information in **Table 4**, representing details about the value and appropriateness of using patient-specific versus uniform policies depends on the costs, efficacies, and the predictive power of the classifier derived from data. Improvements in the accuracy of classifiers lead to greater selectivity in the application of programs and greater overall benefits to patients and hospitals. For example, by using the patient-specific decision analysis in the application of an intervention that costs $1,300 and that is 35% effective, 3.8% of rehospitalization costs can be saved and 18.2% of readmissions can be prevented. However, applying the intervention to all patients does not save dollars. Rather, providing the program to all patients adds 3.2% to the total costs. Intervention decisions obtained using LACE in lieu of the learned classifier would achieve only 0.5% savings and prevented only 2.6% of readmissions.

**Figure 3:**
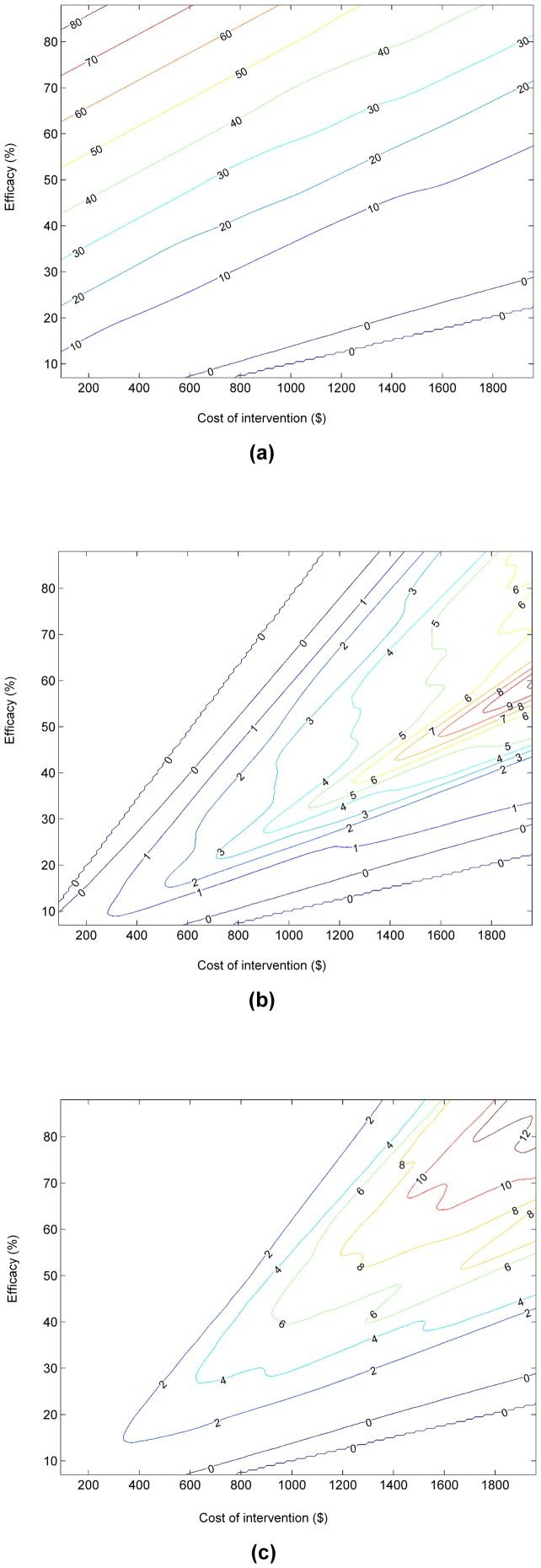
Contour maps capturing cost savings for ranges of program costs and efficacies: (a) savings with decision analysis over no intervention; (b) savings achieved with automated decision analysis over that of applying best uniform policy; (c) savings achieved with automated decision analysis over the use of LACE score, highlighting value of using more accurate predictive model. The labels on contour maps show percentage savings.

Given the ranges of costs and efficacies of post-discharge programs reported in the literature, the efficacy of a specific new program may not be known at the time that a new program is formulated. To build insights about the value of employing a real-time decision system in light of such uncertainty, we introduce an automated analysis of the *net expected value of a decision system* in a clinical environment. For each of a large number of different pairs of assumed efficacy and cost of intervention, we apply the data to prediction to action pipeline for all patients in the validation cohort, using the hospital-specific classifier that we have generated from local data. This sensitivity analysis provides the overall reduction in readmissions or savings for each pair of independently varied cost and efficacy. We performed this analysis and combined the multiple studies into a contour map visualization of the expected savings across the spectrum of cost and efficacy of the intervention. The contour map in **Figure 3**(a) represents the expected reductions in costs achieved with the use of a real-time decision system that performs guidance on patient-specific enrollment versus performing no special intervention for different combinations of cost and efficacies of programs. **Figure 3**(b) considers the boost in savings achieved by using the patient-specific decision guidance over the best uniform policy. The best uniform policy refers to the intervention being performed for all patients or none of the patients, depending on which outcome is better. The contour map demonstrates that the use of a real-time decision-analytic system for guidance would not yield net savings when the intervention is inexpensive and efficient, as it is best in that situation to apply the intervention to the entire population. Likewise, decision-analytic guidance would not be useful when the intervention is very expensive and inefficient, as such a program would not be cost-effective for any patients. However, for interventions with more intermediate costs and efficacies, relative savings of nearly 10% can be realized for the population we have studied, given predictions available from the classifier. **Figure 3**(c) considers the same boost in cost savings achieved by the patient-specific decision analysis over the use of the LACE score. The use of the poorer-performing LACE score for identifying patients at risk for readmissions translates to significantly lower savings and narrower regimes of applicability across regimes defined by the costs and efficacies of programs.

## Discussion and Conclusions

### Implications for readmission reduction

As demonstrated by the results displayed in **Table 4** and **Figure 3**, selective patient enrollment based on automatic risk stratification, could be applied effectively to take advantage of interventional programs that are too expensive to be applied to the entire population of heart failure patients.

The value of performing patient-specific decision analyses will generally increase with increases in classification accuracy, which can be achieved with larger training sets as well as more complete medical information about individual patients. A readmission prediction study [25] conducted in a Veterans Health Administration (VA) Hospital considered a heart failure patient cohort of *n* = 198,460, including data from a cross-hospital EHR containing the long-term medical histories of patients. Using this more extensive data, a statistical classifier was created with an AUC of 0.82. We found that 47% of the readmission patients in our cohort were readmitted to outside medical facilities within 30 days of their initial hospitalization for heart failure. We found that we had sparse EHR data on this substantial proportion of patients and that this may have detracted from the predictive power of our model. The results of the VA study highlight the value of implementing a shared patient database among the different hospitals that patients visit over time.

### Analysis and insights enabled by the electronic capture of clinical data

We see the construction and use of predictive models that are custom tailored to populations as having the ability to deliver predictions of higher accuracy than those produced by simpler clinical rules designed for application across all hospitals. We envision that the *data to prediction to action* pipeline will become more widely used as EHR systems become more prevalent. We now outline several benefits of using local patient data to construct classifiers to predict outcomes, as compared to relying on simpler rules such as LACE.

### Automated risk calculation

The classifier for predictions is constructed entirely from data available within the EHR. In real-time clinical use, findings on specific patients can be drawn automatically from the EHR, requiring no additional time from the practitioners. The computed risk scores can in turn be used to recommend enrollment into prevention programs as part of the workflow. Currently, such enrollment is performed typically using a pencil-and-paper form, or an application specifically designed for this purpose, and observations about patients and their health are manually entered or checked, making it difficult to consider multiple influencing factors. The risk estimation methods we describe do not place additional demands on practitioners and enable large numbers of patients to be screened for multiple intervention and prevention programs. Such automation also enables valuable data on the efficacy of interventions to be collected and harnessed. Moreover, when data on outcomes associated with interventions is accrued in the EHR, we can extend the methodology described in this paper to estimate both the readmission risk and the likelihood of the intervention succeeding at the level of an individual patient or patient subpopulation, further amplifying the effect of the intervention program.

### Emphasis on local data

The predictive model is constructed entirely using local data. If our aim is to make the best possible predictions, taking local populations, data collection, and practice patterns into account will almost always be important in achieving the highest predictive performance. This raises the question of how a model trained within one hospital would perform when validated at a different hospital. As readmission patterns and prevalence vary by hospital and by region [26], a risk-stratification model that is not constructed locally will not be able to take into account the location-specific trends, details of care activities, and patient population. Unlike a generic risk rule, a locally trained classifier will be able to adjust as changes in the hospital's patterns of care occur over time.

### Clinical face validity of observations

In reviewing the most predictive variables found by the classifier, we found that low lymphocyte percentage (top variable in **Table 2**) has previously been linked to poor outcomes in early postdischarge period of hospitalized HF patients [27] and low hematocrit (second top variable in **Table 3**) has been related to lower risk of heart failure [28]. However, a benefit of the data-driven approach is that we make use of all available data in constructing a predictive model. This means that we do not restrict the analysis solely to variables that have been found valid in previous studies. This characteristic is important because a learning algorithm may derive significant predictive power from variables that have poor face validity and appear arbitrary. This predictive power can stem from correlations among seemingly irrelevant variables available in the EHR and other, more interpretable variables that are not captured in the patient record. Variables identified as discriminatory may serve as proxies for other variables that would be more understandable, but are not available in the system. For example, in one of our studies on predicting general readmissions to the hospital, we discovered that “cocaine test: negative” raises the likelihood that the patient will be readmitted. A subsequent inquiry revealed that clinicians would be unlikely to administer a cocaine screening unless they had reason to suspect that a patient may be a drug abuser. Thus, the observation that the test was administered at all is linked to a higher-level assessment by the physician that the patient belongs to a vulnerable population—an assessment that is not recorded directly in the health database. Classifiers derived from all of the data available in the EHR will generally perform as well or better than models restricted only to those variables that have widely recognized clinical significance or that mesh with intuition as understandable or obvious indicators of risk with respect to a specific target ailment. We note that great care must be taken in interpreting the clinical relevance of variables flagged by such models as discriminating. Such observations are not necessarily involved in a *causal* manner nor suggest new paths to intervention. Observations identified as useful by the learning procedure may reflect local care patterns and not necessarily correspond to a generalizable risk factor. At the same time, variables identified as discriminating for a given predictive task may unearth new clinical insights and frame directions for study and intervention.

### On limitations of local predictive tools

A potential drawback of the analytical pipeline that we have presented versus traditional risk-stratification scores is that its implementation would require some amount of computing resources. As the approach is data driven and relies only on data within the EHR, it can be implemented by the hospital, by the EHR vendor, or by an independent third party, further lowering the cost of implementing such a predictive system.

Another limitation of this study is the fact that it is based on retrospective data. A more rigorous validation would entail performing a randomized controlled experiment. However, the results provide a first step to demonstrate the applicability of risk stratification for guiding allocation of post-discharge interventions in a cost-effective manner.

## Supporting Information

Text S1 **Supplemental Details to Materials and Methods.**
(PDF)
