# Supplementary material for: Data-Driven Decisions for Reducing Readmissions for Heart Failure: General Methodology and Case Study
Source: PLoS One. 2014 Oct 8;9(10):e109264. doi: 10.1371/journal.pone.0109264 (PMC4190088; doi:10.1371/journal.pone.0109264)
Supplement: Text S1 — Supplemental Details to Materials and Methods. (PDF) [file pone.0109264.s001.pdf]

Text S1. Supplemental Details to Materials and Methods

Outside hospital readmissions

A significant challenge with drawing data from the EHR of a single hospital to construct predictive models and to calculate readmissions is the lack of data about patient visits to other medical facilities. According to CMS, incentives and penalties for readmissions, care, and follow-up for patients admitted for heart failure are the responsibility of the admitting hospital, even when the patient is readmitted to another hospital. We found that a significant portion of patients in the study were readmitted to outside hospitals. Building a predictive model solely based on local EHR data uses incorrect data about valid readmissions. We obtained the correct value for bb_{30} in our cohort via CMS data files, which indicate if a readmitted patient is readmitted by the home hospital or to another hospital. We found that patients who are readmitted to other hospitals tend to receive ongoing care at other hospitals and, thus, outside visits on readmission are associated with gaps in the historical data captured in the local EHR. We refer to these patients as *remote patients* and studied removing these patients from the training cohort to avoid biasing at training time. We denote the updated cohort as the *pruned derivation*, consisting of 725 visits, versus the *full derivation* cohort with 793 visits. We tested the performance of using the pruned derivation as the training set versus using the full derivation through a cross-validation analysis that is explained below.

Cross-validation analysis

In order to finalize construction of the predictive model, we had to make two decisions: (i) selecting the best tuning parameter λ for LASSO; (ii) selecting the pruned versus the full training cohort. The optimal decision was obtained using a leave-one-out twentyfold cross-validation analysis by assigning each of the visits in the full derivation cohort randomly to one of twenty groups of roughly equal size. Then, each of the twenty groups was used as a holdout set for validating and the classifier that was trained on the visits from the remaining nineteen groups and the area under the ROC (AUC) or c-statistics for predicting on the holdout set was recorded. The AUC for the values of λ between 0 and 50 was calculated, once with the pruned derivation and once with the full derivation, for each of the twenty instances and the average AUC was recorded. The optimal decisions for both (i) and (ii) correspond to the largest average AUC.

Comparison with LACE

The LACE [1] score can be calculated from length of stay, acuteness of admission, emergency department visits, and comorbidity score. We calculated all these variables from data stored within the local EHR. In particular, we followed authors of [1] to calculate the comorbidity score. Default implementation of LACE produces a *total score* that is an integer between 0 and 19. We followed the guideline suggested by the authors of [1] to transform the total score to a probability using $p = 1/[1+\exp(-a \cdot L - b)]$, where p is the probability, L is the LACE score, and a, b are two constants. In order to obtain a, b a logistic regression is trained on derivation cohort

where LACE score is the only predictor and the binary response variable is bb_{30}. This results in coefficient a for the LACE score and an intercept term b.

Calibration and reclassification

We performed a calibration study to test whether the probabilistic output of the classifiers was close to outcome probabilities for patients grouped by risk. The calibration is achieved through a similar procedure used for transforming the LACE score to probability. In particular, we used $p = 1/[1 + \exp(-c \cdot P - d)]$, where P is the predicted probability, p is the classifier's final calibrated output, and constant terms c, d are obtained via training a logistic regression on the full derivation set where P is the only predictor and the response variable is bb_{30}.

To check the accuracy of the calibration, all CHF visits in the derivation and validation sets were separately divided into three subgroups, based on the predicted probability of readmission after calibration. The patient subgroups were indexed by "Low risk," "Moderate risk," and "High risk" by values of probability of readmission. We then compared the observed and expected readmission rates for patients in each subgroup. The thresholds for these three groups were obtained by dividing the histogram of log likelihood of predicted probabilities in derivation cohort into three groups of nearly equal size.

We produced a similar grouping of patients using the LACE score and then calculated the reclassification of each patient from the three risk groups identified by LACE to the classifier's three risk groups in the derivation cohort.

REFERENCES

1. Van Walraven C, Dhalla I. A., Bell Ch, Etchells E, Stiell IG et al. (2010), Derivation and validation of an index to predict early death or unplanned readmission after discharge from hospital to the community. CMAJ. 6:51–557.
